# Advancing the Global Fight Against HIV/Aids: Strategies, Barriers, and the Road to Eradication

**DOI:** 10.5334/aogh.4277

**Published:** 2023-11-27

**Authors:** Emmanuel Kumah, Dorothy Serwaa Boakye, Richard Boateng, Eunice Agyei

**Affiliations:** 1 Department of Health Administration and Education, Faculty of Science Education, University of Education, Winneba, Ghana

**Keywords:** HIV/AIDS, epidemic, global response strategies, barriers

## Abstract

HIV/AIDS remains one of the most significant global health challenges, affecting millions of people worldwide. Since the inception of the disease, various global response strategies have been devised and implemented, aiming to mitigate its impact and ultimately eradicate it. While these strategies have yielded remarkable progress, there are still key barriers impeding the global fight against the disease. This paper, thus, delves into the key global response strategies employed in response to the HIV/AIDS epidemic since its inception, examines the impediments to their successful implementation, and outlines the trajectory towards a world without AIDS. To continue the momentum in the fight against HIV/AIDS, it is imperative to adopt a multifaceted approach that addresses the existing barriers. One pivotal aspect of this approach involves intensifying efforts to improve the uptake of HIV testing. Encouraging individuals to get tested is a critical step, as it not only aids in identifying more cases of HIV infection but also facilitates the linkage of those affected to appropriate care and support services.

## Introduction

The global HIV/AIDS epidemic has been one of the most significant public health challenges of the past few decades [1]. First identified in the early 1980s, HIV (human immunodeficiency virus) is a virus that attacks the body’s immune system, specifically targeting CD4+ T cells, which are crucial for maintaining a functional immune response [2]. As the virus replicates and destroys these cells, the body becomes progressively more susceptible to opportunistic infections and certain types of cancer. When left untreated, HIV infection can lead to the development of AIDS (acquired immunodeficiency syndrome), a condition in which the immune system is severely compromised, and life-threatening illnesses can occur [2].

The disease has affected millions of people worldwide, with sub-Saharan Africa (SSA) being the most heavily impacted region [1]. According to the Joint United Nations Program on HIV/AIDS (UNAIDS), as of 2022, approximately 39 million people were living with HIV globally. Of these, an estimated 25.6 million were in SSA, accounting for nearly two-thirds of all HIV infections [3]. Other regions, such as Asia, Latin America, and Eastern Europe, have also seen significant increases in HIV prevalence, though not on the same scale as SSA. The disease has had particularly devastating effects on the people of SSA, affecting not only individuals but also economies and social structures [3].

## Global response to the HIV/AIDS epidemic

The global response to the HIV/AIDS epidemic has been a critical area of focus for the international community for several decades. This has been characterized by international collaboration, advocacy, and resource mobilization to tackle the multifaceted challenges posed by the disease. For instance, UNAIDS, a joint program of the United Nations dedicated to addressing the disease, was established in 1996 to coordinate international efforts, advocate for increased funding and resources, and support countries in implementing effective HIV/AIDS programs [4]. Improving access to HIV treatment, particularly through the widespread availability of antiretroviral therapy (ART), has also been a significant focus of the world response to HIV/AIDS. The World Health Organization (WHO), for instance, introduced the “Treat All” policy in 2015, recommending immediate initiation of ART for all people living with HIV [5]. Another crucial aspect of the global response to HIV/AIDS is prevention. The UNAIDS strategy in this area focuses on combination prevention, including measures such as condom distribution, harm reduction programs for injecting drug users, and promoting voluntary medical male circumcision in high-prevalence regions [6]. The WHO’s Option B+ strategy, which involves providing lifelong ART to all pregnant and breastfeeding women living with HIV, has been widely adopted as a strategy to prevent mother-to-child transmission of HIV. Pre-Exposure Prophylaxis (PrEP) has also emerged as a critical prevention tool for people at high risk of HIV infection [5]. PrEP is a highly effective method for individuals at high risk of HIV infection, as it involves taking antiretroviral medication to reduce the likelihood of contracting the virus [5]. Other strategies that have been employed to combat the HIV/AIDS epidemic include addressing stigma and discrimination, integrating HIV services with other health interventions, empowering key population groups, and investing in research and innovation [6, 7].

## Impact of the global HIV/AIDS response strategies

The global response strategies have resulted in significant progress in the global fight against HIV/AIDS. For instance, the UNAIDS’ 2022 report indicates that since 2010, annual new HIV infections have declined by 38%, from 2.1 million to 1.3 million in 2022. AIDS-related deaths have also decreased by 69% and 51% since 2004 and 2010, respectively, dropping from 2 million and 1.3 million to 630,000 in 2022 [8]. And among all people living with HIV, 86% know their status, 89% are accessing treatment, and 93% are virally suppressed [3]. This represents significant progress toward attaining the new 95-95-95 global target of diagnosing 95% of HIV-positive individuals, providing ART for 95% of those diagnosed, and achieving viral suppression for 95% of those treated by 2025 [8]. Overall, the US President’s Emergency Plan for AIDS Relief (PEPFAR) estimates that in the past three decades, access to HIV treatment has averted about 20.8 million AIDS-related deaths worldwide [9]. PEPFAR is a funding organization initiated by the US government to combat the global HIV/AIDS epidemic. It was launched by President George W. Bush in 2003 and has been a significant contributor to the international effort to prevent and treat HIV/AIDS. PEPFAR provides financial support to countries heavily impacted by the HIV/AIDS epidemic, aiming to improve access to prevention, care, and treatment services [10].

## Barriers to implementing global HIV/AIDS interventions

While there have been positive outcomes, several barriers persist, which hamper the global fight against the HIV/AIDS epidemic. Access to HIV testing and ART has been a critical issue in the global fight against the disease. Despite significant progress in raising awareness about HIV/AIDS and improving healthcare infrastructure, substantial challenges remain in ensuring widespread and equitable access to testing and treatment. In 2022, about 9.2 million people living with HIV were not receiving treatment, while about 5.5 million people did not know they were living with the condition [3]. Many low- and middle-income countries (LMCs) continue to face barriers to obtaining life-saving medications [6].

Another key barrier to the global effort in the fight against HIV/AIDS pertains to stigma and discrimination. This negative social perception toward people living with HIV/AIDS is having devastating consequences for individuals and communities. It is hindering HIV prevention programs by deterring individuals from getting tested, disclosing their status, and practicing safer behaviors [11].

Gender inequality is also playing a substantial role in increasing the vulnerability of women and girls to HIV infection. Societal norms, unequal power dynamics, and violence against women are hindering their ability to negotiate safe sex and access healthcare [12]. In 2022, for instance, women and girls accounted for 63% of all new HIV infections in the WHO African region [3].

Furthermore, insufficient sex education and a lack of awareness about safe sexual practices, particularly in developing countries, have become significant impediments to the global fight against HIV/AIDS [13]. Several barriers hinder efforts to improve sex education and awareness in developing countries. Sociocultural taboos around discussing sexuality, along with limited resources for educational initiatives, pose challenges to implementing effective programs [13].

Moreover, insufficient funding for HIV/AIDS programs and research remains a significant barrier to progress in combating the epidemic [3,13]. A 2022 report by amfAR (The Foundation for AIDS Research) indicates that declining investment in research has resulted in fewer scientific breakthroughs and a slowdown in the development of new antiretroviral drugs and preventive measures [14]. This lack of financial support stifles innovation and prolongs the timeline for achieving a world free from AIDS.

## Achieving an AIDS-free world: the way forward

The future direction in the fight against HIV/AIDS should be marked by a commitment to a comprehensive and inclusive approach that addresses the individual, social, and structural factors contributing to the epidemic. It also involves harnessing the power of science, technology, and community involvement to reduce new infections, improve the quality of life for those living with HIV, and ultimately work towards ending the HIV/AIDS epidemic.

Of particular importance is intensifying efforts to improve the uptake of HIV testing. HIV testing plays a pivotal role as the primary and indispensable component of the HIV cascade of care, acting as the crucial first step in managing the epidemic [15]. It represents the first of the three goals set by UNAIDS to achieve epidemic control by 2025, where 95% of people living with HIV will be aware of their status [16].

Accurate and readily accessible HIV testing is of paramount importance. This accessibility is a cornerstone of directing individuals who test positive for HIV to receive the vital treatment they need [15]. Prompt linkage to care and treatment significantly improves the health and quality of life for those living with HIV, reducing death rates and enhancing overall well-being [15]. Furthermore, by identifying new cases and providing early access to treatment, we can reduce the viral load in the global community, thereby lowering the risk of transmission. A study by Cohen et al., published in the New England Journal of Medicine, found that early initiation of ART reduced the risk of HIV transmission to uninfected partners by a remarkable 93% [17].

One pivotal aspect of HIV testing lies in its profound influence on high-risk behaviors. Evidence shows that knowing one’s status is closely associated with reductions in behaviors that carry a high risk of transmission [18]. Such behaviors include syringe sharing among people who inject drugs and inconsistent condom use among those engaged in sexual activities [18]. Being aware of one’s HIV/AIDS status acts as a catalyst for safer practices, as individuals are more likely to adopt protective measures when they understand the potential risks involved. This, in turn, contributes to curbing the transmission of the virus and decreasing the burden of the epidemic [18].

In pursuit of this goal, the WHO has taken an active role in advocating for and promoting HIV testing as a key strategy in the global fight against the HIV/AIDS epidemic. One of the foundational documents outlining the WHO’s approach to HIV testing is the “Consolidated Guidelines on HIV Testing Services,” which was first published in 2015 and updated in 2019 [19]. These guidelines emphasize the importance of making HIV testing accessible, available, and acceptable to all while respecting individual autonomy and rights. The guidelines highlight the benefits of testing, including early diagnosis, timely treatment initiation, and prevention of HIV transmission [19].

The WHO advocates for a range of HIV testing approaches, including facility-based testing, community-based testing, self-testing, and partner notification [19]. These approaches are tailored to the diverse needs and preferences of individuals in different settings. For instance, the promotion of self-testing allows individuals to test for HIV in the privacy of their own homes, which can help reduce stigma and increase testing uptake.

Furthermore, the WHO underscores the importance of integrating HIV testing with other health services, such as sexual and reproductive health services, tuberculosis screening, and harm reduction programs for people who inject drugs [19]. This integration could help normalize HIV testing and reach individuals who might otherwise not seek testing services.

In promoting HIV testing, the WHO also emphasizes the importance of ensuring informed consent, confidentiality, and linkage to care and support services for those who test positive. These principles are outlined in various WHO documents, including the “Guidelines on HIV Self-Testing and Partner Notification [19].”

It is worth noting that the success of HIV testing programs also relies on addressing structural barriers such as stigma, discrimination, and legal obstacles [20]. The WHO recognizes the need for policy and legal environments that support testing for HIV and protect the rights of people living with HIV/AIDS [19].

## Conclusion

The global HIV/AIDS epidemic remains a significant public health challenge, particularly in sub-Saharan Africa and other vulnerable regions. While progress has been made in treatment and prevention, addressing issues of access, funding, stigma, and discrimination remains crucial to achieving effective control and eventual eradication of the disease. Promoting testing for HIV remains an essential strategy in the fight against the epidemic. Testing initiates the cascade of care, linking individuals to treatment and prevention services, which, in turn, facilitates viral load suppression and reduces the spread of the disease. The WHO has, therefore, been actively involved in advocating for and promoting HIV testing as a key strategy in the global response to the HIV/AIDS epidemic.

## Data Accessibility Statements

All relevant data are within the paper.
